# Preparation of Retinoyl-Flavonolignan Hybrids and Their Antioxidant Properties

**DOI:** 10.3390/antiox8070236

**Published:** 2019-07-23

**Authors:** Christopher S. Chambers, David Biedermann, Kateřina Valentová, Lucie Petrásková, Jitka Viktorová, Marek Kuzma, Vladimír Křen

**Affiliations:** 1 Laboratory of Biotransformation, Institute of Microbiology of the Czech Academy of Sciences, Vídeňská 1083, 14220 Prague, Czech Republic; 2 Department of Biochemistry and Microbiology, University of Chemistry and Technology, Technická 5, 16628 Prague, Czech Republic

**Keywords:** carotenoids, retinol, retinoic acid, vitamin A, flavonolignans, silymarin, antioxidant, anti-radical, esterification, conjugate

## Abstract

Antioxidants protect the structural and functional components in organisms against oxidative stress. Most antioxidants are of plant origin as the plants are permanently exposed to oxidative stress (UV radiation, photosynthetic reactions). Both carotenoids and flavonoids are prominent antioxidant and anti-radical agents often occurring together in the plant tissues and acting in lipophilic and hydrophilic milieu, respectively. They are complementary in their anti-radical activity. This study describes the synthesis of a series of hybrid ester conjugates of retinoic acid with various flavonolignans, such as silybin, 2,3-dehydrosilybin and isosilybin. Antioxidant/anti-radical activities and bio-physical properties of novel covalent carotenoid-flavonoid hybrids, as well as various mixtures of the respective parent components, were investigated. Retinoyl conjugates with silybin—which is the most important flavonolignan in silymarin complex—(and its pure diastereomers) displayed better 1,1-diphenyl-2-picrylhydrazyl (DPPH) radical scavenging activity than both the parent compounds and their equimolar mixtures.

## 1. Introduction

Antioxidants are substances that protect the structural and functional components in living organisms—especially proteins, lipid structures (membranes) and nucleic acids—against oxidative stress. Protein damage by oxidative radicals and reactive oxygen species (ROS, e.g., OH^●^, O_3_^●^, NO, ROO^●^, H_2_O_2_, ^1^O_2_, HClO, etc.) leads to partial or complete loss of function and also to the risk of autoimmune diseases due to cross-immune reactivity between the damaged (immunogenic) and intact proteins [1]. Most of the antioxidants are of plant origin as the plants are permanently exposed to oxidative stress (UV radiation, photosynthetic reactions) and cannot escape [2]. Antioxidants mostly act in complex systems in biological structures, often as redox tandems. Neutralizing a highly reactive radical with an antioxidant can lead to another, usually less reactive radical, which can also be dangerous in the case of accumulation. Antioxidants—especially at higher doses—often act as oxidative stressors. Different antioxidant mechanisms of complementary antioxidants thus act much more effectively than single antioxidants even at a higher concentration. Cocktails of various antioxidants are often the key ingredients of various nutraceuticals claiming to protect against oxidative stress. The most important antioxidants include a number of vitamins (ascorbate, retinol and tocopherol) and prominent bioactives such as carotenoids and flavonoids [3]. Nevertheless, the concept of antioxidants as active redox species in vivo has been re-evaluated as a number of “antioxidants” actually act as weak prooxidants or they directly interact with some nuclear receptors, such as NF-κB, thus regulating the production of intracellular enzymatic antioxidant systems [4].

**Figure 1:**
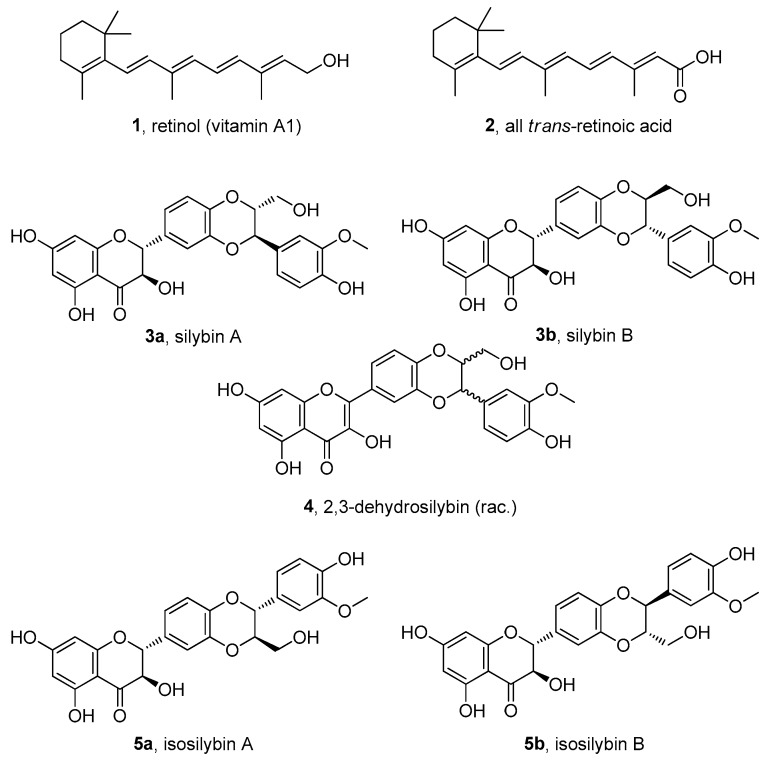
Structures of retinol, retinoic acid and selected flavonolignans from *Silybum marianum* (L.) Gaertn. (Asteraceae). Note to the numbering: Natural silybin is extracted and often used as an approximately equimolar diastereomeric mixture of silybin A (**3a**) and silybin B (**3b**). Diastereomeric mixtures of silybin and its derivatives will be denoted with the respective number and letters **ab** (for example natural mixture of **3a** and **3b** will be denoted **3ab**).

Carotenoids [5] and flavonoids [6] are natural products that are found throughout the plant kingdom and play a crucial role in photoreception, photoprotection, antioxidant processes and some of them—namely carotenoids—in photosynthesis [2]. They are also very important as exogenous biologically active substances (vitamins) for animals and humans and are commonly consumed in the diet (Figure 1).

Combining antioxidants having different mechanisms of action in a single molecule often leads to a strong potentiation of the antioxidant activity. This approach has already been used in hybrids containing, for example, tocopherol and procaine [7]; iron chelator deferiprone (3-hydroxy-2-methyl-4(1*H*)-pyridinone) and 3,5-disubstituted-4-hydroxyphenyl derivatives like BHT (butylated hydroxytoluene) [8], flavonolignans and fatty acids [9] or ascorbic acid [10] with improved antioxidant or other specific properties. Carotenoids have been rarely used in this manner probably due to their limited stability. Astaxanthin has been esterified with ferulic acid to obtain astaxanthin diferulate, improving the radical scavenging ability [11]. 4′-Hydroxyflavone has also been combined with 12′-apo-β-carotenal, which resulted in the formation of C–C linked artificial carotenylflavonoid [12,13]. Flavonoids and especially their glycosides, are considerably more hydrophilic than carotenoids. Interactions of flavonoids with carotenoids often occur at the water/lipid interfaces. The activity of flavonoids appears to be more pronounced in the aqueous phase, whereas carotenoids are more active in the lipid phase [14].

The aim of the present study was to synthetize a series of hybrid conjugates of retinol (**1**) and/or retinoic acid (**2**) with various flavonolignans such as silybin A (**3a**), silybin B (**3b**), 2,3-dehydrosilybin (**4**), isosilybin A (**5a**) and isosilybin B (**5b**) from silymarin. (Figure 1) The biophysical properties of supramolecular conjugates of these two important types of antioxidants were investigated.

## 2. Materials and Methods

### 2.1. Chemicals

Retinol (**1**) and retinoic acid (**2**) were obtained from Sigma-Aldrich (Prague, Czech Republic). Silybin diastereomeric mixture (**3ab**), optically pure silybins A, B (**3a**, **3b**) [9] and isosilybin A (**5a**) [15] were prepared from silymarin purchased from Liaoning Senrong Pharmaceutical Co. (Panjin, People’s Republic of China, batch no. 120501) according to the published procedures. 2,3-Dehydrosilybin (**4**) was prepared from silybin as previously described [16].

### 2.2. Nuclear Magnetic Resonance (NMR) and Mass Spectrometry (MS) Methodology

A Bruker Avance III 700 MHz spectrometer (700.13 MHz for ^1^H, 176.05 MHz for ^13^C) and Bruker Avance III 600 MHz spectrometer (600.23 MHz for ^1^H, 150.93 MHz for ^13^C, Bruker BioSpin, Rheinstetten, Germany) were used for NMR analysis of samples in dimethylsulfoxide (DMSO)-*d*_6_ (99.8% atom D, VWR International, Stříbrná Skalice, Czech Republic) at 30 °C. The residual signals of the solvent were used as internal standards (*δ*_H_ 2.499, *δ*_C_ 39.46). The following experiments were performed and processed using the manufacturer’s software (Topspin 3.2, Bruker BioSpin, Rheinstetten, Germany): ^1^H NMR, ^13^C NMR, ge-2D homonuclear correlation spectroscopy (COSY), ge-2D multiplicity-edited ^1^H–^13^C heteronuclear single-quantum correlation spectroscopy (HSQC), ge-2D ^1^H–^13^C heteronuclear multiple-bond correlation spectroscopy (HMBC) and 1D total correlation spectroscopy (TOCSY). ^1^H NMR and ^13^C NMR spectra were zero filled to four-fold data points and multiplied by a window function before the Fourier transformation.

The signal-to-noise ratio in ^13^C NMR spectra was improved by application of a line broadening (1 Hz). The resolution in ^1^H NMR spectra were accomplished by the two-parameter double-exponential Lorentz–Gauss function applied prior to Fourier transformation. Multiplicity-edited ^1^H-^13^C HSQC spectra were utilized to identify the multiplicity of the ^13^C signals. Chemical shifts are reported in *δ*-scale. The digital resolution of spectra allowed us to report carbon chemical shift to two decimal places and hydrogen chemical shifts to three decimal places.

The LTQ Orbitrap XL hybrid mass spectrometer (Thermo Fisher Scientific, Waltham, MA, USA) equipped with an electrospray ion source was used to measure high resolution mass spectra (HRMS). The samples dissolved in MeOH were introduced into the mobile phase flow (MeOH/H_2_O 4:1; 100 μL/min) using a 2 μL loop. Spray, capillary and tube lens voltage were 4.0 kV, −16 V, −120 V, respectively; capillary temperature was 275 °C.

### 2.3. HPLC

The Shimadzu Prominence System (Shimadzu Corporation, Kyoto, Japan) consisting of a DGU-20A3 mobile phase degasser, two LC-20AD solvent delivery units, a SIL-20AC cooling auto sampler, a CTO-10AS column oven and SPD-M20A diode array detector was employed for analytical high performance liquid chromatography (HPLC) analyses. The Chromolith Performance RP-18e monolithic column (100 mm × 3 mm i.d., Merck, Darmstadt, Germany) coupled with a guard column (5 mm × 4.6 mm i.d., Merck, Darmstadt, Germany) was used. Mobile phase acetonitrile (phase A) and water (phase B) were used for the analyses; gradient: 0 80% B; 20 min 0% B, 22–24.5 min 80% B at 25 °C; the flow rate was 1.0 mL/min. The Photometric Diode Array (PDA) data were acquired in the 200–450 nm range and signals at 360 nm were extracted. Chromatographic data were collected and processed using Shimadzu Solution software (version 5.75 SP2, Shimadzu Corporation, Tokyo, Japan) at a rate of 40 Hz and detector time constant of 0.025 s.

Preparative HPLC separations were carried out with a Shimadzu system consisting of a LC-8A high-pressure pump with a SPD-20A dual wavelength detector (with preparative cell) and FRC-10A fraction collector. The system was connected to a PC using CBM-20A command module and controlled by a LabSolution 1.24 SP1 software suite (all Shimadzu, Kyoto, Japan). All preparative HPLC separations were performed using an ASAHIPAK GS-310 20F column (Shodex, Munich, Germany) at 25 °C with MeOH as mobile phase, flow rate 5 mL/min and detection at 254 and 369 nm.

### 2.4. Chemical Synthesis

#### 2.4.1. General Procedures for the Synthesis of Conjugates

Method A: Retinoic acid (**2**, 0.5 mmol, 1 eq), flavonolignan (**3**–**5**, 0.75 mmol, 1.5 eq), *N,N*′-dicyclohexylcarbodiimide (DCC, 1 mmol, 2 eq) and 4-dimethylaminopyridine (DMAP, catalytic amount) were dissolved in dry tetrahydrofuran (THF, 25 mL). The reaction mixture was stirred at room temperature (25 °C) in darkness under argon for 18 h. The solvent was removed in vacuo and the crude mixture was separated by silica gel chromatography (19:1; CHCl_3_/acetone). Dicyclohexylurea (DCU, a by-product of the Steglich reaction) was removed by preparative HPLC.

Method B: Retinoic acid (**2**, 0.3 mmol, 1.5 eq), flavonolignan (**3**–**5**, 0.2 mmol, 1 eq), *N*-ethyl-*N*′-(3-dimethylaminopropyl)carbodiimide hydrochloride (EDC·HCl, 0.4 mmol, 2 eq) and DMAP (catalytic amount) were dissolved in dry THF (9 mL) and *N,N*-dimethylformamide (DMF, 1 mL). After stirring for 5 h in darkness under argon, the reaction mixture was diluted with EtOAc and the organic layer was separated and washed with water (2 × 15 mL). The combined organic layers were dried (Na_2_SO_4_), filtered and the solvent was removed in vacuo. Purification was performed by preparative HPLC and the fractions containing the product were further purified by silica chromatography (CHCl_3_ 100% to CHCl_3_/acetone; 19:1).

The reactions were monitored by thin layer chromatography (TLC) on silica gel plates (9:2:0.1; CHCl_3_/acetone/formic acid).

#### 2.4.2. Synthesis of Conjugates

*Silybin-7*-O-*retinoate* (**6ab**): Natural silybin (**3ab**) and retinoic acid (**2**) were reacted according to the method A or method B to yield title compound **6ab** as yellow solid; method A (32 mg, 0.042 mmol, 13.2%); method B (22 mg, 0.028 mmol, 9.6%). For ^1^H and ^13^C NMR data see Table S1 in the Supplementary Material. HRESIMS *m/z*: [M − H]^−^ calcd for C_45_H_47_O_11_ 763.31239; found 763.31182 (Figure S1).

*Silybin A-7*-O-*retinoate* (**6a**): Silybin A (**3a**) and retinoic acid (**2**) were reacted according to the method A or method B to yield title compound **6a** as yellow solid; method A (62 mg, 0.081 mmol, 25%); method B (23 mg, 0.030 mmol, 10%). For ^1^H and ^13^C NMR data see Table S2 in the Supplementary Material. HRESIMS *m/z*: [M − H]^−^ calcd for C_45_H_47_O_11_ 763.31239; found 763.31127 (Figure S2).

*Silybin B-7*-O-*retinoate* (**6b**): Silybin B (**3b**) and retinoic acid (**2**) were reacted according to the method A or method B to yield title compound **6b** as yellow solid; method A (76 mg, 0.099 mmol, 31%); method B (44 mg, 0.058 mmol 19.2%). For ^1^H and ^13^C NMR data see Table S3 in the Supplementary Material. HRESIMS *m/z*: [M − H]^−^ calcd for C_45_H_47_O_11_ 763.31239; found 763.31138 (Figure S3).

*2,3-Dehydrosilybin AB-3*-O-*retinoate* (**7**): Racemic 2,3-dehydrosilybin (**4**) and retinoic acid were reacted according to the method A or method B to yield title compound **7** as yellow solid; method A (52 mg, 0.068 mmol, 21%); method B (49 mg, 0.064 mmol, 21%). For ^1^H and ^13^C NMR data see Table S4 in the Supplementary Material. HRESIMS *m/z*: [M − H]^−^ calcd for C_45_H_45_O_11_ 761.29674; found 761.29657 (Figure S4).

*Isosilybin A-7*-O-*retinoate* (**8a**): Isosilybin A (**5a**) and retinoic acid (**2**) were reacted according to the method A or method B to yield title compound **8a** as yellow solid; method A (58 mg, 0.076 mmol, 24%); method B (57 mg, 0.075 mmol, 25%). For ^1^H and ^13^C NMR data see Table S5 in the Supplementary Material. HRESIMS *m/z*: [M − H]^−^ calcd for C_45_H_47_O_11_ 763.31239; found 763.31197 (Figure S5).

*Isosilybin B-7*-O-*retinoate* (**8b**): Isosilybin B (**5b**) and retinoic acid (**2**) were reacted according to the method A to yield title compound **8b** as yellow solid (23 mg, 0.030 mmol, 9.5%). For ^1^H and ^13^C NMR data see Table S6 in the Supplementary Material. HRESIMS *m/z*: [M − H]^−^ calcd for C_45_H_47_O_11_ 763.31239; found 763.31207 (Figure S6).

The purity of all compounds used for consecutive tests was over 91% as determined by HPLC.

### 2.5. Antioxidant Activity

#### 2.5.1. Determination of Log P Values

The lipophilicity/hydrophilicity of the compounds (miLogP) was calculated using the Molinspiration property engine v2016.10 (http://www.molinspiration.com, Molinspiration Cheminformatics, Slovensky Grob, Slovakia) [17].

#### 2.5.2. DPPH Radical Scavenging

The radical scavenging activity of the prepared conjugates, their parent compounds, their equimolar mixtures and retinol was evaluated as their capacity to scavenge 1,1-diphenyl-2-picrylhydrazyl radicals (DPPH, Sigma-Aldrich, Prague, Czech Republic) [18], as described previously [10] with minor modifications. Briefly, 15 µL of the tested substance (final concentration 0–20 mM in DMSO) was mixed with 285 µL of a freshly prepared methanolic DPPH solution (final concentration 20 µM) in a microtiter plate well. After 30 min at 25 °C, the absorbance at 517 nm was measured. The activity was expressed as the concentration of the compound required for reducing the absorbance to 50% (IC_50_) of its initial value.

#### 2.5.3. Antioxidant Activity

Ferric reducing antioxidant power (FRAP) [19] and cupric reducing antioxidant capacity (CUPRAC) [20] were measured using kits from Bioquochem (Llanera-Asturias, Spain) according to the manufacturer´s instructions and expressed as trolox equivalents (TE). To determine oxygen radical absorption capacity (ORAC) [21], 73 µL of fluorescein (1.8 mg/L) solution in phosphate buffered saline (PBS, pH 7.4) was mixed with 2 µL of the samples (0–20 µM) in the wells of a 96-well plate. After 15 min incubation (37 °C), 25 µL of freshly prepared 2,2′-azo-*bis*(2-amidinopropane) dihydrochloride (AAPH, 60 mg/mL) was added to each well except for the negative control, where AAPH was replaced for PBS. The fluorescence (ex./em., 485/535 nm) was recorded immediately and for the next 2 h with the measurement step for 5 min using a microplate reader (SpectraMax i3 Multi-Mode Detection Platform, Molecular Devices, Wokingham, UK).

For cellular antioxidant activity (CAA) assay [22], the HepG2 cell line (ATCC, CCL-23TM, USA) was cultivated in Eagle’s Minimum Essential Medium (Sigma-Aldrich, USA) supplemented with 10% of foetal bovine serum, 2 mM l-glutamine and 1× Antibiotic Antimycotic Solution (Sigma-Aldrich, St. Louis, MO, USA) in a CO_2_ incubator (5% CO_2_, 37 °C, Thermo Fisher Scientific, Waltham, MA, USA) and passaged 2×/week using trypsin-EDTA solution. For the experiments, 100 µL of the cells (1 × 10^6^ cells/mL, Cellometer Auto T4 Bright Field Cell Counter, Nexcelom Bioscience, Lawrence, MA, USA) were split into 96-well plates. After 24 h, the cells were washed 3× with PBS (MultiFlo Multi-Mode Dispenser, BioTek, USA) and Dulbecco’s Modified Eagle’s Medium (Sigma-Aldrich, St. Louis, MO, USA) supplemented with the 2′,7′-dichlorodihydrofluorescein diacetate (DCFH-DA, 0.0125 mg/mL) was added to each well together with the tested samples in the concentration range 0–500 µM. After incubation (1 h, CO_2_ incubator, 37 °C), the medium was manually replaced for AAPH solution (0.16 mg/mL) and the fluorescence (ex./em. 485/540 nm) was recorded immediately for 2 h with 5 min steps.

#### 2.5.4. Statistical Analysis

All data were analysed with one-way ANOVA, Scheffé and least squares difference tests for post hoc comparisons among pairs of means using the statistical package Statext ver. 2.1 (STATEXT LLC, Wayne, NJ, USA). Differences were considered statistically significant when *p* < 0.05.

## 3. Results and Discussion

### 3.1. Synthesis of Conjugates

Due to the inherent sensitivity of carotenoids and flavonoids to oxidation under harsh conditions in organic syntheses, an enzymatic approach using the lipase Novozym 435^®^ with a cross linker was first tested for the linking of carotenoids with flavonoids. Such procedures have the advantage of being chemo-, regio- and in some cases even stereoselective while working in mild conditions [23] and Novozym 435^®^ is highly selective for primary alcohol groups [10]. This lipase or various oxidases were previously used to prepare dimers of silybin (**3ab**) [24] and 2,3-dehydrosilybin (**4**), as well as heterodimers [25], combined with different flavonoids, which showed interesting antioxidant and biological activities. Also, retinol (**1**) was conjugated with dicarboxylic acids, lactate and oleate under lipase catalysis to reduce photodestruction and possible irritation when used in cosmetics [26]. By chemical synthesis, a set of dimers of various carotenoid-linked dicarboxylates was prepared [27]. Water-soluble carotenoid conjugates with hydrophilic polyols linked by dicarboxylic linkers were prepared using lipase [28]. An interesting heterodimer of retinol was prepared by chemically catalysed esterification of retinol (**1**) and retinoic acid (**2**) [29]. There are many examples of coupling retinol (**1**) and its derivatives to palmitic acid to make a long aliphatic ester [30].

**Scheme 1:**
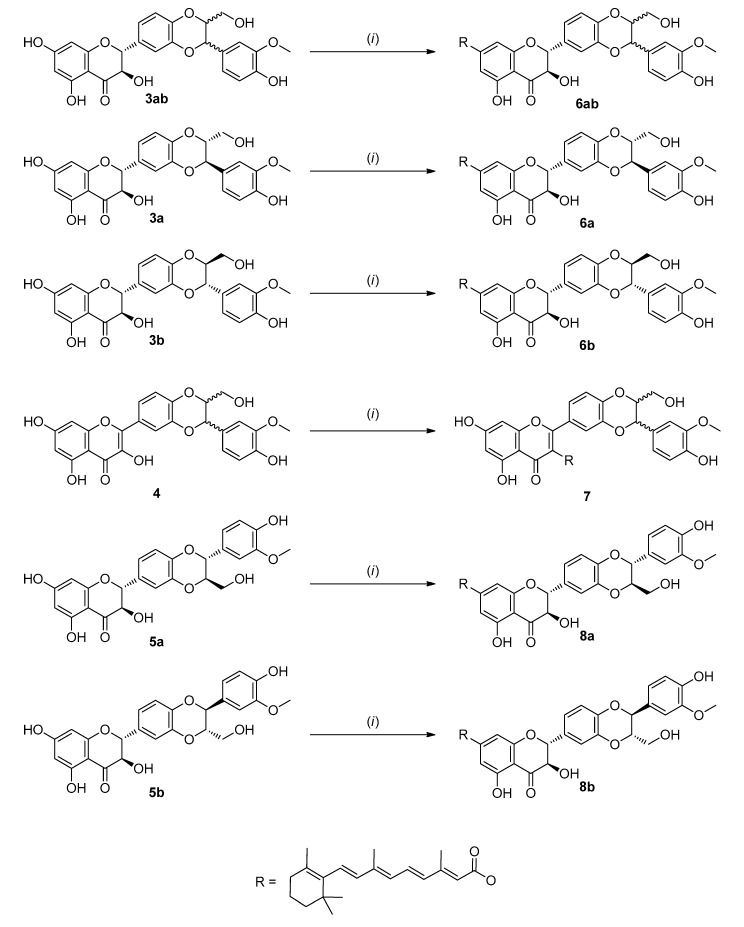
Esterification of the flavonolignans with retinoic acid. (*i*) Method A: **2**, DCC, DMAP, THF, r.t., 18 h or Method B: **2**, EDC·HCl, DMAP, THF, 5 h.

However, the enzymatic method proved to be unfeasible for the conjugation of retinol with the flavonolignans **3ab**, **3a**, **3b**, **4**, **5a** and **5b** and some previously published enzymatic methods for retinol conjugation [28] failed to be reproduced in our hands. Therefore, an original, so far unexplored chemical approach for the ester linkage formation was chosen. The classic Steglich approach was utilized using DCC and a catalytic amount of DMAP (Scheme 1). The reactions proceeded to yield the derivatives with the retinoic moiety at the C-7 position of the flavonolignan except for 2,3-dehydrosilybin where the 3-*O*-retinyl-2,3-dehydrosilybin was isolated as the major product. During Steglich reaction a by-product DCU was formed, which could be removed using ASAHIPAK GS-310 20F column, a hitherto unknown procedure for DCU removal. Unfortunately, during the separation of the products the parent flavonolignan also co-eluted with the respective product. The product was then purified by silica chromatography in darkness. To overcome the contamination problem EDC·HCl was used, which could easily be removed by washing with water.

All newly prepared carotenoyl-flavonolignans were fully characterized by ^1^H and ^13^C NMR and their structures were confirmed using HRMS. In general, the isolated yields are just moderate ranging from 9–25%, which was caused partly by decomposition of the starting material and often by formation of rather complex reaction mixtures, which were not quite easy to separate even using preparative HPLC. This esterification was regioselective providing nearly exclusively 7-*O*-esters of flavonoids, which is a great advantage as it avoids tedious protection/deprotection procedures of multifunctional (five OH groups) flavonolignan structure. Only in the case of 2,3-dehydrosilybin the 3-*O*-ester was preferentially formed probably due to higher reactivity of C-3 OH in 2,3-dehydroflavonolignans [10]. Our new method enabled to obtain sufficient amounts of heteroconjugates for antioxidant and further biophysical tests.

### 3.2. Antioxidant and Biophysical Testing of Conjugates

The oxidations in living organism caused by free radicals are usually undesirable processes damaging such important biomolecules as DNA, proteins and lipids [31]. The application of antioxidants is one of the most straightforward means to protect these biomolecules from oxidative stress. Many assays are available for determination of antioxidant potential in vitro. These assays are based on the reduction of stable radicals (DPPH), on the competitive bleaching of a probe (ORAC) or on the reduction of iron ions (CUPRAC, FRAP) [32]. CUPRAC test is very similar to FRAP assay but is based on the reduction of Cu^2+^ in a neutral pH, while FRAP test is based on Fe^3+^ in acidic pH. As each assay suffers from some limitations, a set of antioxidant tests was used here to combine more parameters.

DPPH test is one of the most commonly performed antioxidant assays with natural and (semi)synthetic biologically active compounds. Although it has no direct physiological relevance, this assay allows quick comparison of free radical scavenging potential of new derivatives as this activity has been published for many compounds [33]. We have therefore tested our conjugates for DPPH scavenging and compared their activity with the activity of the parent compounds, that is, retinoic acid and the respective flavonolignan and their equimolar mixtures; retinol (**1**) was used as a benchmark. As expected, the activity of retinoic acid (**2**) was about half that of retinol (**1**) with IC_50_ values 745 and 1485 µM, respectively. In accordance with our previously published data [25,34,35,36] also parent flavonolignans **3ab**, **3a**, **3b** and **5a** displayed relatively low activity (IC_50_ 472–818 µM), while 2,3-dehydrosilybin (**4**) was much more active (19.2 µM, Table 1). The conjugates **6a** and **6b** with silybin diastereomers displayed significantly better DPPH scavenging activity (IC_50_ 379 and 540 µM) than both the parent compounds and their equimolar mixtures. In contrast, the conjugates **6ab**, **7** and **8a** with natural silybin, 2,3-dehydrosilybin and isosilybin A were significantly worse scavengers than the parent compounds and their equimolar mixtures (Table 1). We could only speculate on the reasons why diastereomerically pure conjugates have better antioxidant activity than their equimolar mixture. Nevertheless, even though the differences are significant, the IC_50_ values of all silybin isomers and conjugates are in the same order of magnitude and similar to that of retinol and isosilybin, which all are relatively weak antioxidants. The biological relevance of such differences is probably rather low.

The main disadvantage of DPPH scavenging assay is the use of an unnatural radical [37]. Such methods based on competitive probe reactions or indirect methods based on persistent radicals should only be used for preliminary screening purposes [37]. Thus, to better characterize the potential of the obtained conjugates in terms of their biological activity, a series of simple, rapid, sensitive and reproducible biochemical antioxidant assays including FRAP, CUPRAC and ORAC [38] and a more relevant cellular antioxidant activity (CAA [37]) was measured (Table 2). Many samples having the ability to reduce radicals in chemical assays were shown to fail in cellular assays, which also take into account the bioavailability and first-pass metabolism of tested compounds [39]. Therefore, in this study, we compared two methods with the same mechanism of action: ORAC assay was performed as a classical biochemical method presenting the ability of tested compounds to quench peroxyl radicals generated from AAPH to protect fluorescein from oxidation. This assay serves better as a physical description of the tested compounds [37]. To reflect biological aspect, CAA was measured using the liver carcinoma HepG2 cell line where the compound must enter the cell to fulfil its role as an antioxidant. The CAA assay partially includes the bioavailability, aspects of uptake and metabolism. Despite the mentioned advantages, the CAA does not provide the insight into the fate of tested compounds in the whole organism including distribution, clearance and for example, the ability of tested compounds to induce the transcription of antioxidant enzymes [37].

In most of these tests all the conjugates displayed significantly lower activity than their respective parent compounds. In the CUPRAC assay intended specifically for hydrophobic antioxidants, where in some cases the conjugates **6ab** and **6b** had an activity comparable to the parent flavonolignans, most samples containing a carotenoid moiety precipitated in the reaction mixture thus hampering the activity determination and making the comparison mostly impossible. Dose-dependent curves were obtained in both ORAC and CAA assay for single compounds resulting in IC_50_ determination (Table 2). In contrast, no anti-oxidant activity of the conjugates was detected in liver cells in all the concentration range tested up to 50 or 500 µM. In fact, such a large concentration is not expectable in plasma. Furthermore, the conjugate **6a** proved to be unstable and decomposed during ORAC and CAA measurement.

Diastereomerically pure conjugates **6a** and **6b** displayed significantly better DPPH scavenging activity (IC_50_ 379 and 540 µM) than both the parent compounds and their equimolar mixtures. This finding could have potential exploitation as silybin is the amplest and largely used flavonoid from the silymarin complex. New, so far undescribed, conjugates of retinoic acid and flavonolignans have obviously comparable or even worse antioxidant activity in relation to their parent molecules. We could speculate that one reason could be the blocking of the highly (anti-radical) active [40] moiety at C-7 of flavonolignans and/or substantial increase of lipophilicity of the conjugates (log*P* 7.53 and 8.21, Table 2). Highly lipophilic conjugates are likely to be incorporated into the cell membrane and do not pass into the cells to exert their effect topically in the membrane.

## 4. Conclusions

We have described to our best knowledge the first synthesis of a series of hybrid ester conjugates of retinoic acid with various flavonolignans (flavonoid-carotenoid supramolecular conjugates) such as silybin, 2,3-dehydrosilybin and isosilybin. Antioxidant/anti-radical activities and bio-physical properties of novel carotenoid-flavonoid hybrids as well as various mixtures of the respective components were investigated. The conjugates with silybin diastereomers, which are the most important flavonolignans in silymarin complex, displayed better DPPH scavenging activity than both the parent compounds and their equimolar mixtures. Other conjugates have comparable or even worse antioxidant activity in relation to their parent molecules.

## Figures and Tables

**Table 1:** 1,1-Diphenyl-2-picrylhydrazyl (DPPH) radical scavenging activities (IC_50_ [µM]) of retinoic conjugates in comparison with their parent compounds and their equimolar mixtures.

	Parent Compound	Conjugate with Retinoic Acid	Mixture 1:1 Eq with Retinoic Acid
Retinol (**1**)	745 ± 11	−	−
Retinoic acid (**2**)	1485 ± 110	−	−
Silybin (**3ab**)	472 ± 16	(**6ab**) 666 ± 16 *	499 ± 6
Silybin A (**3a**)	818 ± 22	(**6a**) 379 ± 19 *	750 ± 33
Silybin B (**3b**)	659 ± 29	(**6b**) 540 ± 24 *	773 ± 7
2,3-Dehydrosilybin (**4**)	19.2 ± 0.3	(**7**) 734 ± 35 *	15.1 ± 0.3
Isosilybin A (**5a**)	783 ± 9	(**8a**) 2361 ± 152 *	610 ± 18

Results are presented as means ± standard error from at least three independent experiments. * Values are significantly different (*p* < 0.05) compared to the parent flavonolignan, retinoic acid and their equimolar mixture.

**Table 2:** Antioxidant activities and lipophilicity/hydrophilicity of retinoic conjugates in comparison with their parent compounds.

	FRAP [TE] ^a^	CUPRAC ^b^ [TE]	ORAC ^c^ (IC_50_ [µM])	CAA ^d^ (IC_50_ [µM])	Log*P* ^e^
Retinol (**1**)	1.76 ± 0.04	0.09 ± 0.01^f^	169 ± 12	1271 ± 147	5.92
Retinoic acid (**2**)	0.62 ± 0.02	0.10 ± 0.02^f^	13 ± 2	460 ± 211	5.80
Silybin (**3ab**)	0.335 ± 0.006	0.17 ± 0.00	7.8 ± 0.7	11.8 ± 0.3	1.47
Silybin A (**3a**)	0.278 ± 0.005	0.20 ± 0.00	8.5 ± 0.4	10.0 ± 0.7	1.47
Silybin B (**3b**)	0.268 ± 0.009	0.16 ± 0.02	8.0 ± 0.3	6.8 ± 0.5	1.47
2,3-Dehydrosilybin (**4**)	4.06 ± 0.05	0.25 ± 0.00	9.6 ± 0.4	10.9 ± 0.5	2.44
Isosilybin A (**5a**)	0.280 ± 0.006	0.16 ± 0.02	4.3 ± 1.1	>100 ^h^	1.47
Silybin AB-7-*O*-retinoate (**6ab**)	0.03 ± 0.02 *^,#^	0.22 ± 0.01^#^	91 ± 4 *^,#^	>500 ^h^	7.53
Silybin A-7-*O*-retinoate (**6a**)	0.038 ± 0.002 *^,#^	0.21 ± 0.02^#^	9.0 ± 0.7 ^j^	>50 ^g,h^	7.53
Silybin B-7-*O*-retinoate (**6b**)	0.010 ± 0.001 *^,#^	0.04 ± 0.01^f^	230 ± 8 *^,#^	>500 ^g^	7.53
2,3-Dehydrosilybin-3-*O*-retinoate (**7**)	0.034 ± 0.005 *^,#^	0.07 ± 0.02^f^	130 ± 7 *^,#^	>500 ^g^	8.21
Isosilybin A-7-*O*-retinoate (**8a**)	0.022 ± 0.003 *^,#^	0.11 ± 0.01^f^	174 ± 7 *^,#^	>500 ^g^	7.53

Results are presented as means ± standard error from at least three independent experiments. ^a^ Ferric-reducing antioxidant potential (trolox equivalents, TE); ^b^ cupric reducing antioxidant capacity; ^c^ oxygen radical absorption capacity, ^d^ cellular antioxidant activity; ^e^ hydrophobicity of compounds; *^,#^ values significantly different (*p* < 0.05) compared to the parent flavonolignan (*) and retinoic acid (^#^); ^f^ measurement disturbed by the formation of a precipitate in the reaction mixture; ^g^ the sample decomposed; ^h^ no activity noted at the highest concentration tested.

## Supplementary Materials

Supplementary material containing MS, ^1^H and ^13^C NMR data of the new compounds can be found at https://www.mdpi.com/2076-3921/8/7/236/s1.

## Abbreviations

AAPH	2,2′-Azo-*bis*(2-amidinopropane) dihydrochloride
BHT	Butylated hydroxytoluene
CAA	Cellular antioxidant activity
CUPRAC	Cupric reducing antioxidant capacity
DCC	*N,N*′-Dicyclohexylcarbodiimide
DCFH-DA	2′,7′-Dichlorodihydrofluorescein diacetate
DCU	Dicyclohexylurea
DMAP	4-Dimethylaminopyridine
DPPH	1,1-Diphenyl-2-picrylhydrazyl radical
EDC	1-Ethyl-3-(3-dimethylaminopropyl)carbodiimide
FRAP	Ferric reducing antioxidant power
NF-κb	Nuclear factor kappa-light-chain-enhancer of activated B cells
ORAC	Oxygen radical absorbance capacity
ROS	Reactive oxygen species
THF	Tetrahydrofuran
