# Supplementary material for: Preparation of Retinoyl-Flavonolignan Hybrids and Their Antioxidant Properties
Source: Antioxidants (Basel). 2019 Jul 23;8(7):236. doi: 10.3390/antiox8070236 (PMC6680806; doi:10.3390/antiox8070236)
Supplement: Supplementary file 1 [file antioxidants-08-00236-s001.pdf]

Supplementary Material

Preparation of Carotenoid-Flavonoid Hybrids and their Properties

Christopher S. Chambers[1], David Biedermann[1], Kateřina Valentová[1], Lucie Petrásková[1], Jitka Viktorová[2], Marek Kuzma[1] and Vladimír Křen[1,*]

1 Laboratory of Biotransformation, Institute of Microbiology of the Czech Academy of Sciences, Vídeňská 1083, CZ 142 20 Prague, Czech Republic; christopher.chambers@biomed.cas.cz (C.S.C.); biedermann@biomed.cas.cz (D.B.); kata.valentova@email.cz (K.V.); kuzma@biomed.cas.cz (M.K.)

2 Department of Biochemistry and Microbiology, University of Chemistry and Technology, Prague, Technická 5, CZ 166 28, Prague; (J.V.)

Czech Republic

* Correspondence: kren@biomed.cas.cz; Tel.: +420-296-442-510

Table of content:

**Figure S1.**	HRMS (ESI$^-$) of compound **6ab**.	2
**Table S1.**	^{1}H and ^{13}C NMR data of compound **6ab**.	3
**Figure S2.**	HRMS (ESI$^-$) of compound **6a**.	5
**Table S2.**	^{1}H and ^{13}C NMR data of compound **6a**.	6
**Figure S3.**	HRMS (ESI$^-$) of compound **6b**.	8
**Table S3.**	^{1}H and ^{13}C NMR data of compound **6b**.	9
**Figure S4.**	HRMS (ESI$^-$) of compound **7**.	11
**Table S4.**	^{1}H and ^{13}C NMR data of compound **7**.	12
**Figure S5.**	HRMS (ESI$^-$) of compound **8a**.	14
**Table S5.**	^{1}H and ^{13}C NMR data of compound **8a**.	15
**Figure S6.**	HRMS (ESI$^-$) of compound **8b**.	17
**Table S6.**	^{1}H and ^{13}C NMR data of compound **8b**.	18

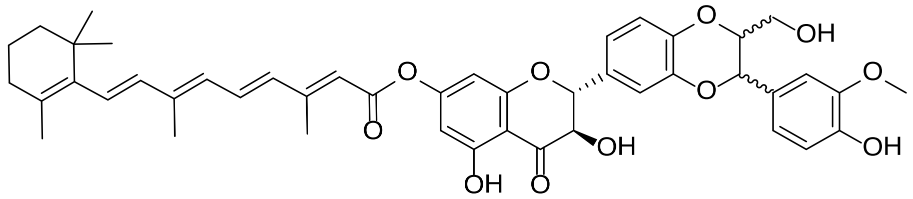

6ab

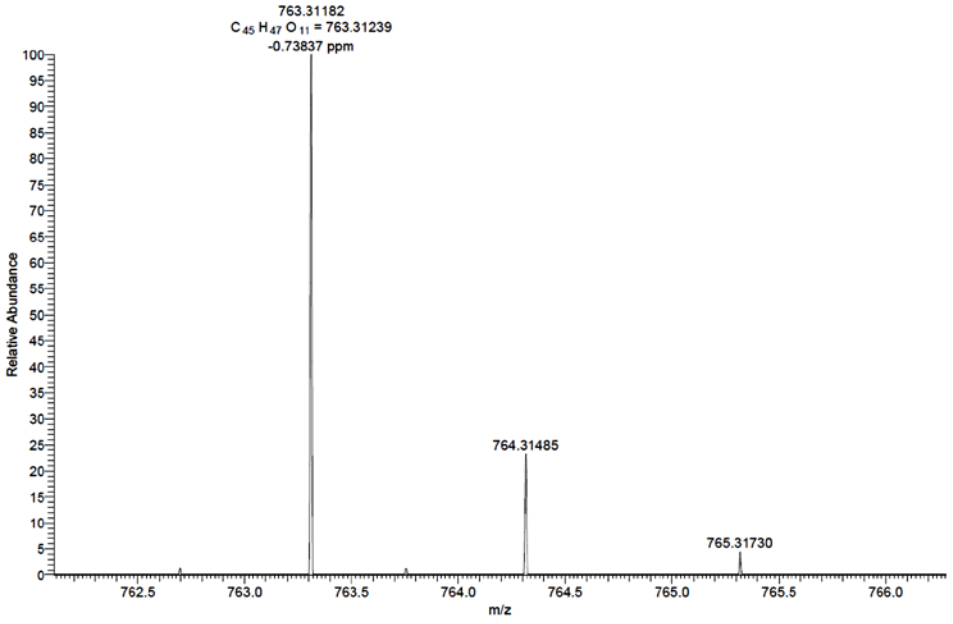

Figure S1. HRMS (ESI$^-$) of compound **6ab** *m/z* [M - H]$^-$ calcd for $C_{45}H_{47}O_{11}$ 763.31239; found 763.31182

Table S1. ^{1}H and ^{13}C NMR data of compound **6ab**.

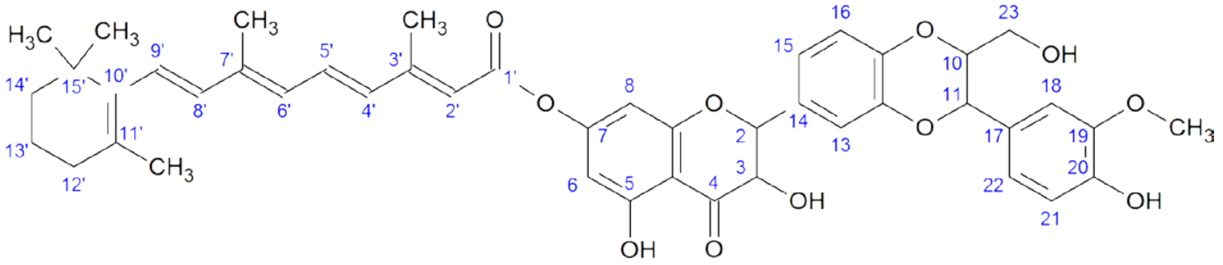

Atom #	δ_C	m	δ_H	n_H	m	$J_{H\text{-}H}$ [Hz]
**2**	82.66 / 82.63	d	5.231 / 5.230	1	d	11.7 / 11.7
**3**	71.60 / 71.67	d	4.782 / 4.771	1	dd	11.7, 6.3 / 11.7, 6.3
**4**	199.39 / 199.37	s	-	-	-	-
**4a**	104.53	s	-	-	-	-
**5**	161.83 / 161.82	s	-	-	-	-
**6**	102.85	d	6.384	1	d	2.0
**7**	158.05	s	-	-	-	-
**8**	101.61	d	6.343 / 6.350	1	d	2.0 / 2.0
**8a**	161.69 / 161.68	s	-	-	-	-
**10**	78.02 / 78.04	d	4.177 / 4.171	1	m	-
**11**	75.75	d	4.916	1	d	7.9
**12a**	143.19	s	-	-	-	-
**13**	116.53 / 116.58	d	7.115 / 7.111	1	d	2.1 / 2.1
**14**	129.57 / 129.62	s	-	-	-	-
**15**	121.31 / 121.18	d	7.035 / 7.037	1	dd	8.4, 2.1 / 8.4, 2.1
**16**	116.24 / 116.28	d	6.983 / 6.984	1	d	8.4 / 8.4
**16a**	143.69 / 143.66	s	-	-	-	-
**17**	127.38	s	-	-	-	-
**18**	111.64 / 111.58	d	7.014 / 7.018	1	d	2.0/ 2.0
**19**	147.53 / 147.54	s	-	-	-	-
**20**	146.93 / 146.92	s	-	-	-	-
**21**	115.22 / 115.20	d	6.803 / 6.801	1	d	8.1 / 8.1
**22**	120.42 / 120.41	d	6.864 / 6.866	1	dd	8.1, 2.0 / 8.1, 2.0
**23**	60.07	t	3.543	1	ddd	12.2, 4.4, 2.6
			3.350	1	ddd	12.2, 6.4, 4.1
**3-OH**	-	-	5.927	1	d	6.3
**5-OH**	-	-	11.657	1	s	-
**19-MeO**	55.60	q	3.777 / 3.778	3	s	-
**20-OH**	-	-	9.110	1	s	-
**23-OH**	-	-	4.925	1	dd	6.4, 4.4
**1'**	163.43	s	-	-	-	-

**2'**	116.03	d	6.052	1	s	-
**3'**	156.44	s	-	-	-	-
**4'**	134.45	d	6.514	1	d	14.9
**5'**	132.90	d	7.190	1	dd	14.9, 11.4
**6'**	129.63	d	6.281	1	d	11.4
**7'**	140.49	s	-	-	-	-
**8'**	136.71	d	6.204	1	d	16.0
**9'**	128.55	d	6.325	1	d	16.0
**10'**	137.14	s	-	-	-	-
**11'**	129.74	s	-	-	-	-
**12'**	32.55	t	2.011	2	m	-
**13'**	18.61	t	1.577	2	m	-
**14'**	39.10	t	1.443	2	m	-
**15'**	33.77	s	-	-	-	-
**3'-Me**	13.84	q	2.343	3	s	-
**7'-Me**	12.61	q	2.015	3	s	-
**11'-Me**	21.42	q	1.695	3	m	-
**15'-Me**	28.71	q	1.021	3	s	-

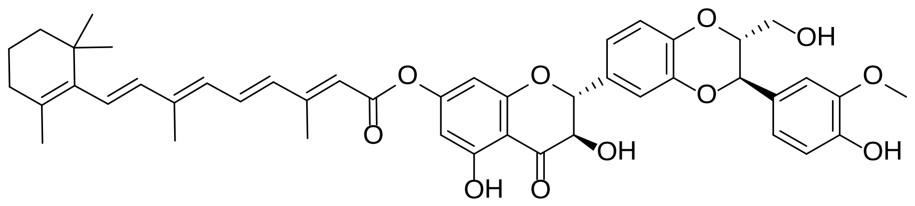

6a

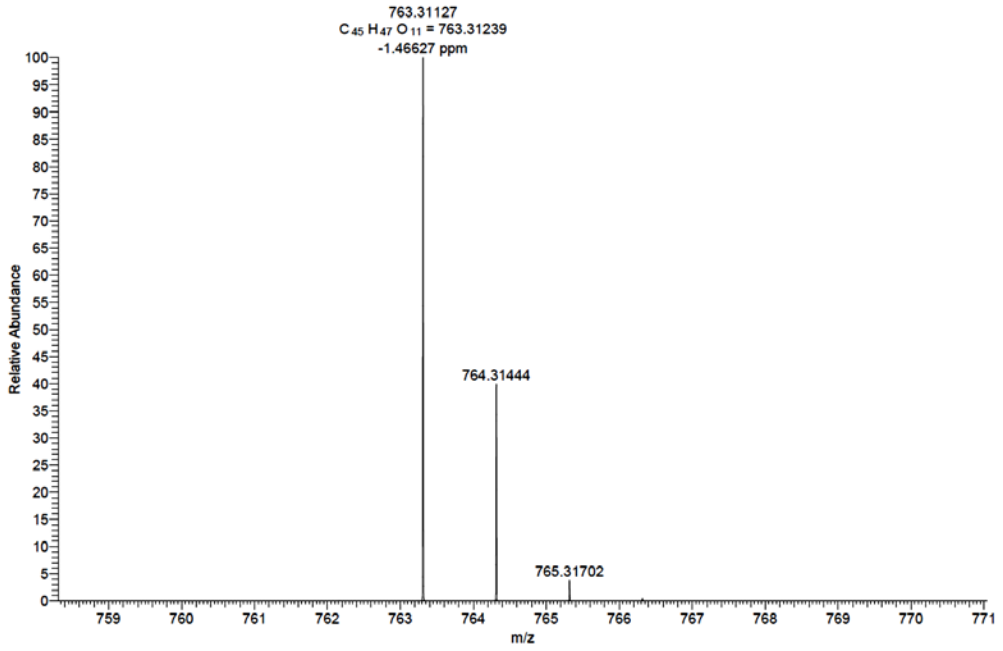

Figure S2. HRMS (ESI$^-$) of compound **6a** *m/z* [M - H]$^-$ calcd for $C_{45}H_{47}O_{11}$ 763.31239; found 763.31127

Table S2. ^{1}H and ^{13}C NMR data of compound **6a**.

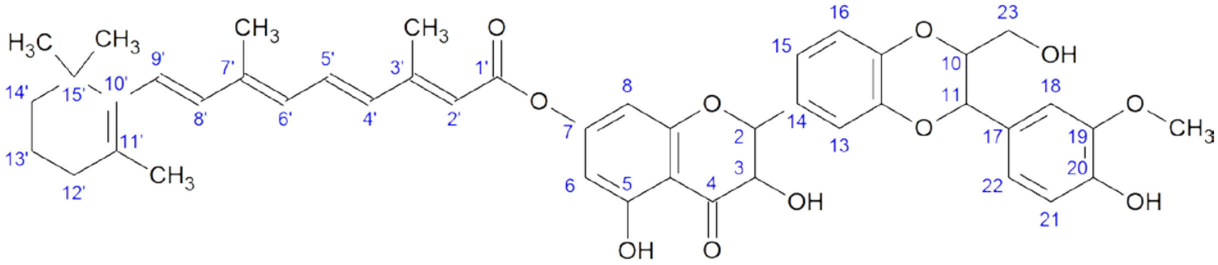

Atom #	δ_C	m	δ_H	n_H	m	$J_{H\text{-}H}$ [Hz]
**2**	82.66	d	5.231	1	d	11.6
**3**	71.60	d	4.782	1	dd	11.6, 6.4
**4**	199.39	s	-	-	-	-
**4a**	104.53	s	-	-	-	-
**5**	161.83	s	-	-	-	-
**6**	102.85	d	6.384	1	d	2.1
**7**	158.05	s	-	-	-	-
**8**	101.61	d	6.343	1	d	2.1
**8a**	161.69	s	-	-	-	-
**10**	78.02	d	4.177	1	ddd	8.0, 4.5, 2.5
**11**	75.74	d	4.916	1	d	8.0
**12a**	143.19	s	-	-	-	-
**13**	116.54	d	7.115	1	d	2.0
**14**	129.57	s	-	-	-	-
**15**	121.31	d	7.035	1	dd	8.3, 2.0
**16**	116.24	d	6.983	1	d	8.3
**16a**	143.69	s	-	-	-	-
**17**	127.37	s	-	-	-	-
**18**	111.64	d	7.014	1	d	1.9
**19**	147.53	s	-	-	-	-
**20**	146.93	s	-	-	-	-
**21**	115.22	d	6.803	1	d	8.1
**22**	120.42	d	6.864	1	dd	8.1, 1.9
**23**	60.08	t	3.543	1	ddd	12.3, 4.6, 2.5
			3.350	1	ddd	12.3, 4.5, 6.1
**3-OH**	-	-	5.926	1	d	6.4
**5-OH**	-	-	11.681	1	s	-
**19-MeO**	55.61	q	3.777	3	s	-
**20-OH**	-	-	9.111	1	s	-
**23-OH**	-	-	4.923	1	dd	6.1, 4.6
**1'**	163.43	s	-	-	-	-

**2'**	116.03	d	6.051	1	s	-
**3'**	156.44	s	-	-	-	-
**4'**	134.45	d	6.513	1	d	15.1
**5'**	132.90	d	7.189	1	dd	15.1, 11.6
**6'**	129.63	d	6.280	1	d	11.6
**7'**	140.49	s	-	-	-	-
**8'**	136.71	d	6.204	1	d	16.0
**9'**	128.55	d	6.324	1	d	16.0
**10'**	137.14	s	-	-	-	-
**11'**	129.74	s	-	-	-	-
**12'**	32.55	t	2.012 [J]	2	m	-
**13'**	18.60	t	1.577	2	m	-
**14'**	39.10	t	1.443	2	m	-
**15'**	33.77	s	-	-	-	-
**3'-Me**	13.84	q	2.342	3	s	-
**7'-Me**	12.61	q	2.015	3	s	-
**11'-Me**	21.42	q	1.695	3	s	-
**15'-Me**	28.71	q	1.021	6	s	-

[J] - *J*-resolved readout

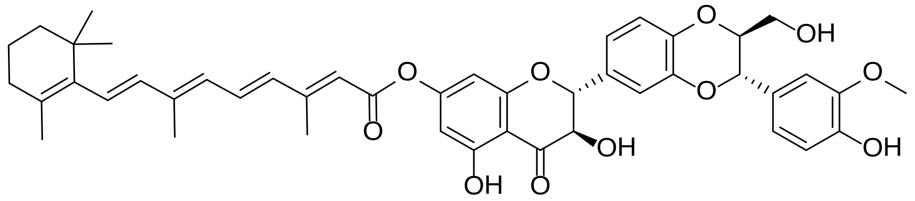

6b

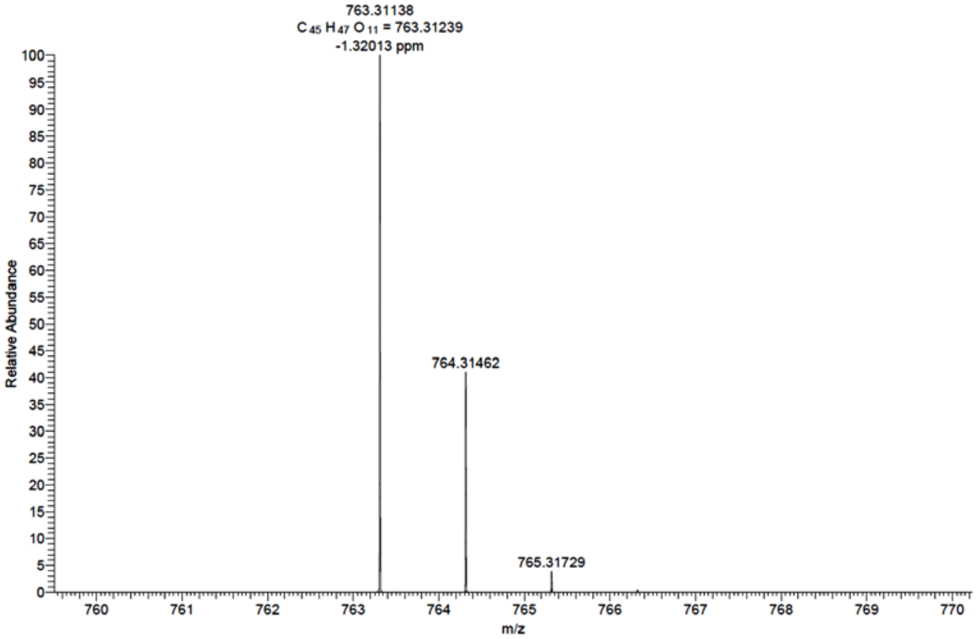

Figure S3. HRMS (ESI⁻) of compound **6b** *m/z* [M - H]⁻ calcd for $C_{45}H_{47}O_{11}$ 763.31239; found 763.31239

Table S3. ^{1}H and ^{13}C NMR data of compound **6b**.

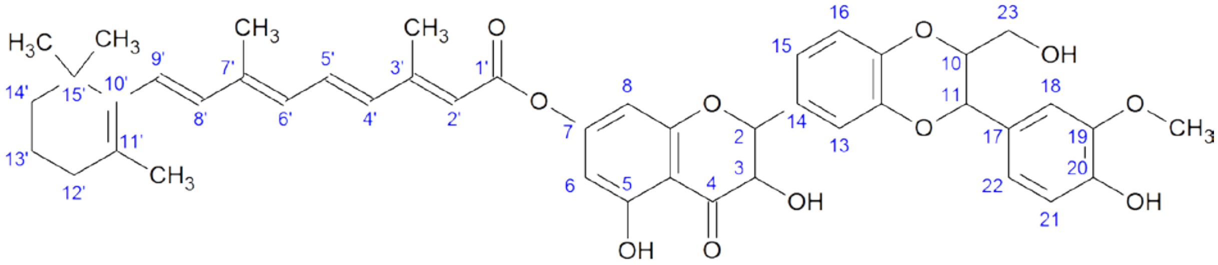

Atom #	δ_C	m	δ_H	n_H	m	J_{H-H} [Hz]
**2**	82.62	d	5.230	1	d	11.6
**3**	71.67	d	4.772	1	dd	11.6, 6.4
**4**	199.37	s	-	-	-	-
**4a**	104.53	s	-	-	-	-
**5**	161.82	s	-	-	-	-
**6**	102.85	d	6.384	1	d	2.0
**7**	158.05	s	-	-	-	-
**8**	101.62	d	6.350	1	d	2.0
**8a**	161.67	s	-	-	-	-
**10**	78.03	d	4.171	1	ddd	7.9, 4.5, 2.5
**11**	75.75	d	4.916	1	d	7.9
**12a**	143.18	s	-	-	-	-
**13**	116.58	d	7.111	1	d	2.0
**14**	129.62	s	-	-	-	-
**15**	121.18	d	7.037	1	dd	8.3, 2.0
**16**	116.28	d	6.985	1	d	8.3
**16a**	143.66	s	-	-	-	-
**17**	127.38	s	-	-	-	-
**18**	111.58	d	7.018	1	d	1.9
**19**	147.54	s	-	-	-	-
**20**	146.92	s	-	-	-	-
**21**	115.20	d	6.800	1	d	8.1
**22**	120.41	d	6.866	1	dd	8.1, 1.9
**23**	60.07	t	3.543	1	ddd	12.3, 5.0, 2.5
			3.349	1	ddd	12.3, 4.5, 6.0
**3-OH**	-	-	5.926	1	d	6.4
**5-OH**	-	-	11.684	1	s	-
**19-MeO**	55.60	q	3.778	3	s	-
**20-OH**	-	-	9.104	1	s	-
**23-OH**	-	-	4.925	1	dd	6.0, 5.0
**1'**	163.43	s	-	-	-	-

**2'**	116.03	d	6.053	1	s	-
**3'**	156.44	s	-	-	-	-
**4'**	134.44	d	6.515	1	d	15.0
**5'**	132.90	d	7.191	1	dd	15.0, 11.4
**6'**	129.63	d	6.281	1	d	11.4
**7'**	140.49	s	-	-	-	-
**8'**	136.71	d	6.205	1	d	16.0
**9'**	128.55	d	6.325	1	d	16.0
**10'**	137.13	s	-	-	-	-
**11'**	129.73	s	-	-	-	-
**12'**	32.55	t	2.014 [J]	2	m	-
**13'**	18.60	t	1.578	2	m	-
**14'**	39.10	t	1.444	2	m	-
**15'**	33.77	s	-	-	-	-
**3'-Me**	13.84	q	2.344	3	s	-
**7'-Me**	12.61	q	2.016	3	s	-
**11'-Me**	21.42	q	1.696	3	s	-
**15'-Me**	28.71	q	1.022	6	s	-

[J] - *J*-resolved readout

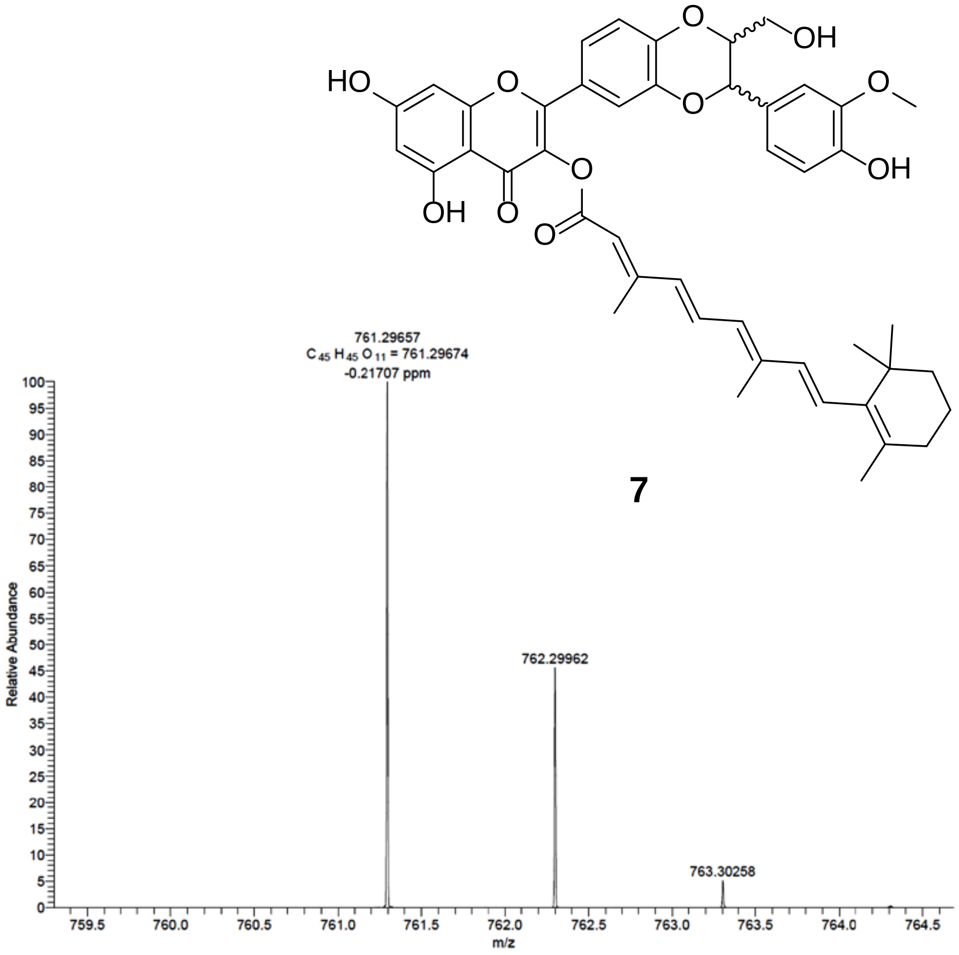

Figure S4. HRMS (ESI$^-$) of compound **7** *m/z* [M - H]$^-$ calcd for $C_{45}H_{45}O_{11}$ 761.29674; found 761.29657

Table S5. ^{1}H and ^{13}C NMR data of compound **7**.

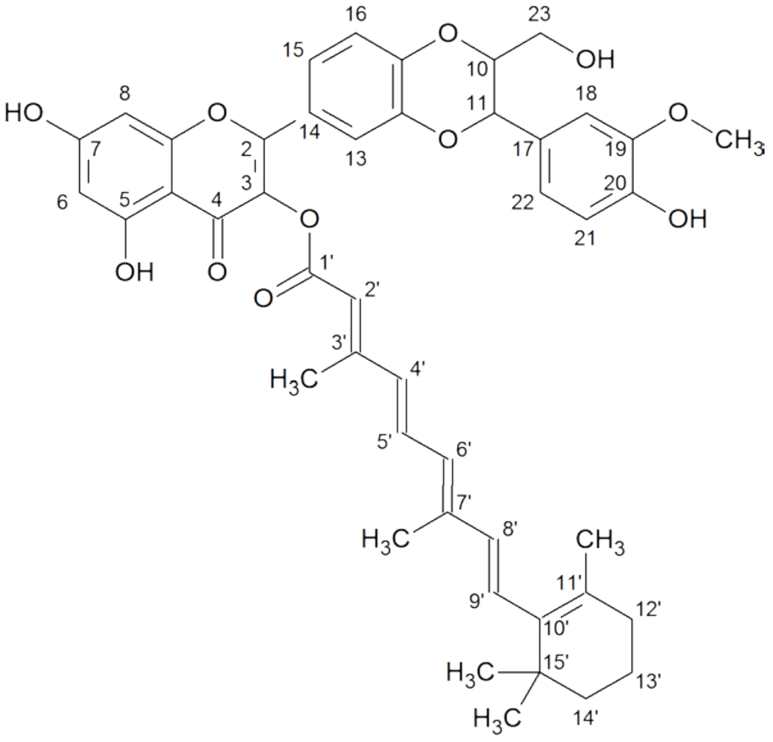

Atom #	δ_C	m	δ_H	n_H	m	$J_{H\text{-}H}$ [Hz]
**2**	155.25	s	-	-	-	-
**3**	130.39	s	-	-	-	-
**4**	175.28	s	-	-	-	-
**4a**	103.59	s	-	-	-	-
**5**	161.25	s	-	-	-	-
**6**	99.33	d	6.233	1	d	2.0
**7**	165.27	s	-	-	-	-
**8**	94.42	d	6.492	1	d	2.0
**8a**	156.88	s	-	-	-	-
**10**	78.72	d	4.269	1	ddd	7.9, 4.8, 2.3
**11**	76.06	d	4.955	1	d	7.9
**12a**	143.88	s	-	-	-	-
**13**	116.66	d	7.461	1	d	2.0
**14**	121.97	s	-	-	-	-
**15**	121.85	d	7.452	1	dd	8.5, 2.0
**16**	117.40	d	7.118	1	d	8.5
**16a**	146.59	s	-	-	-	-
**17**	127.17	s	-	-	-	-
**18**	111.79	d	7.011	1	d	1.8
**19**	147.80	s	-	-	-	-
**20**	147.29	s	-	-	-	-

**21**	115.46	d	6.796	1	d	8.1
**22**	120.72	d	6.863	1	dd	8.1, 1.8
**23**	60.16	t	3.568	1	dd	12.4, 2.3
			3.358	1	dd	12.4, 4.8
**5-OH**	-	-	n.a.	-	-	-
**7-OH**	-	-	n.a.	-	-	-
**19-MeO**	55.72	q	3.761	3	s	-
**20-OH**	-	-	n.a.	-	-	-
**23-OH**	-	-	n.a.	-	-	-
**1'**	163.44	s	-	-	-	-
**2'**	115.41	d	6.154	1	s	-
**3'**	157.30	s	-	-	-	-
**4'**	134.59	d	6.526	1	d	15.0
**5'**	133.38	d	7.185	1	dd	15.0, 11.5
**6'**	129.81	d	6.267	1	d	11.5
**7'**	140.85	s	-	-	-	-
**8'**	136.98	d	6.189	1	d	16.1
**9'**	128.86	d	6.321	1	d	16.1
**10'**	137.37	s	-	-	-	-
**11'**	129.98	s	-	-	-	-
**12'**	32.76	t	1.994	2	m	-
**13'**	18.84	t	1.571	2	m	-
**14'**	39.3[H]	t	1.434	2	m	-
**15'**	33.97	s	-	-	-	-
**3'-Me**	13.94	q	2.317	3	s	-
**7'-Me**	12.67	q	2.003	3	s	-
**11'-Me**	21.54	q	1.689	3	s	-
**15'-Me**	28.83	q	1.011	6	s	-

[H] - HSQC readout

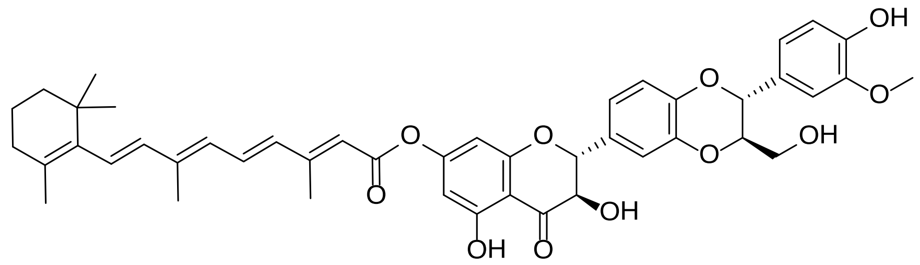

8a

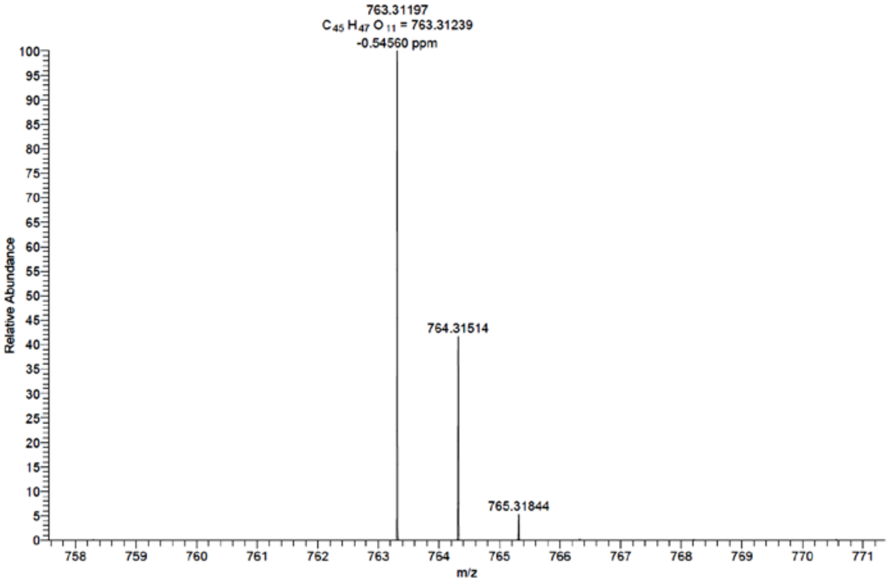

Figure S5. HRMS (ESI$^-$) of compound **8a** *m/z* [M - H]$^-$ calcd for $C_{45}H_{47}O_{11}$ 763.31239; found 763.31197

Table S5. ^{1}H and ^{13}C NMR data of compound **8a**

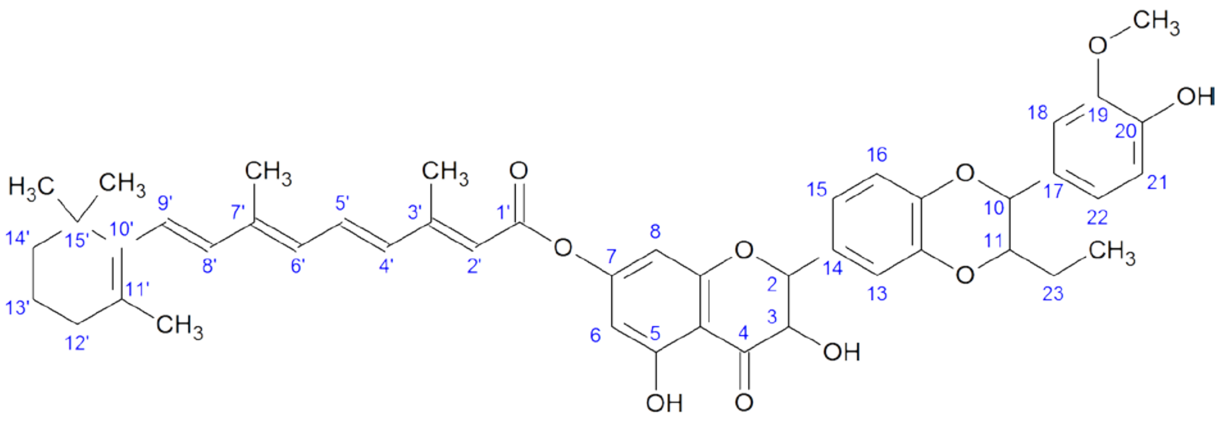

Atom #	δ_C	m	δ_H	n_H	m	J_{H-H} [Hz]
**2**	82.63	d	5.252	1	d	11.5
**3**	71.69	d	4.759	1	dd	11.5, 6.3
**4**	199.25	s	-	-	-	-
**4a**	104.53	s	-	-	-	-
**5**	161.82	s	-	-	-	-
**6**	102.81	d	6.391	1	d	2.0
**7**	158.04	s	-	-	-	-
**8**	101.59	d	6.361	1	d	2.0
**8a**	161.66	s	-	-	-	-
**10**	75.72	d	4.926	1	d	7.6
**11**	77.89	d	4.168	1	ddd	7.6, 4.6, 2.7
**12a**	142.82	s	-	-	-	-
**13**	116.39	d	7.125	1	d	1.8
**14**	129.79	s	-	-	-	-
**15**	120.86	d	7.014	1	dd	8.4, 1.8
**16**	116.34	d	6.945	1	d	8.4
**16a**	143.90	s	-	-	-	-
**17**	127.36	s	-	-	-	-
**18**	111.71	d	7.007	1	d	1.8
**19**	147.49	s	-	-	-	-
**20**	146.91	s	-	-	-	-
**21**	115.24	d	6.806	1	d	8.1
**22**	120.34	d	6.861	1	dd	8.1, 1.8
**23**	60.06	t	3.548	1	ddd	12.2, 4.6, 2.7
			3.357	1	ddd	12.2, 5.6, 4.6
**3-OH**	-	-	5.924	1	d	6.4
**5-OH**	-	-	11.678	1	s	-
**19-MeO**	55.62	q	3.780	3	s	-

**20-OH**	-	-	9.082	1	s	-
**23-OH**	-	-	4.901	1	dd	5.6, 4.6
**1'**	163.40	s	-	-	-	-
**2'**	115.99	d	6.056	1	s	-
**3'**	156.40	s	-	-	-	-
**4'**	134.41	d	6.518	1	d	15.1
**5'**	132.86	d	7.194	1	dd	15.1, 11.6
**6'**	129.70	d	6.284	1	d	11.6
**7'**	140.46	s	-	-	-	-
**8'**	136.66	d	6.206	1	d	16.1
**9'**	128.53	d	6.33 [H]	1	m	-
**10'**	137.11	s	-	-	-	-
**11'**	129.58	s	-	-	-	-
**12'**	32.52	t	2.01 [H]	2	m	-
**13'**	18.58	t	1.582	2	m	-
**14'**	39.11	t	1.448	2	m	-
**15'**	33.74	s	-	-	-	-
**3'-Me**	13.83	q	2.349	3	d	0.8
**7'-Me**	12.57	q	2.018	3	s	-
**11'-Me**	21.37	q	1.698	3	s	-
**15'-Me**	28.68	q	1.024	3	s	-

[H] - HSQC readout

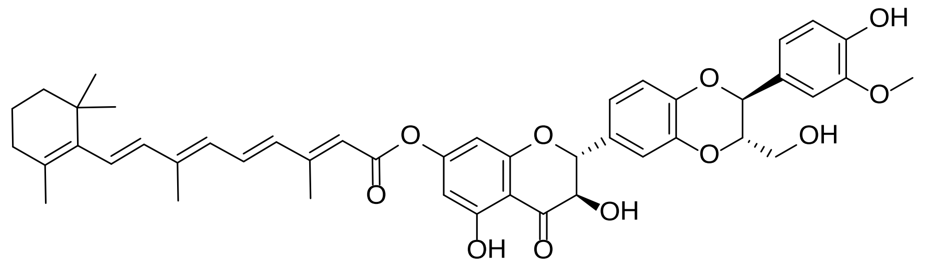

8b

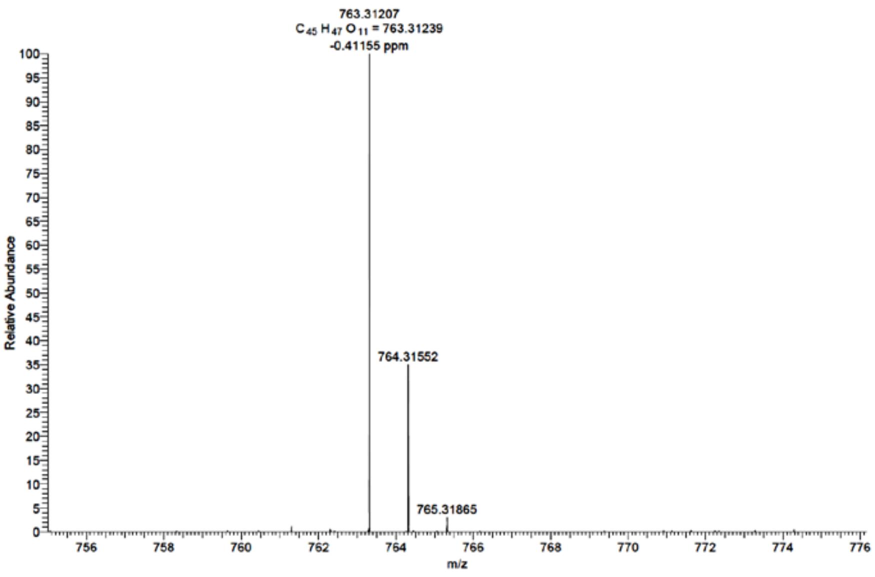

Figure S6. HRMS (ESI$^-$) of compound **8b** *m/z* [M - H]$^-$ calcd for $C_{45}H_{47}O_{11}$ 763.31239; found 763.31197

Table S6. ^{1}H and ^{13}C NMR data of compound **8b**.

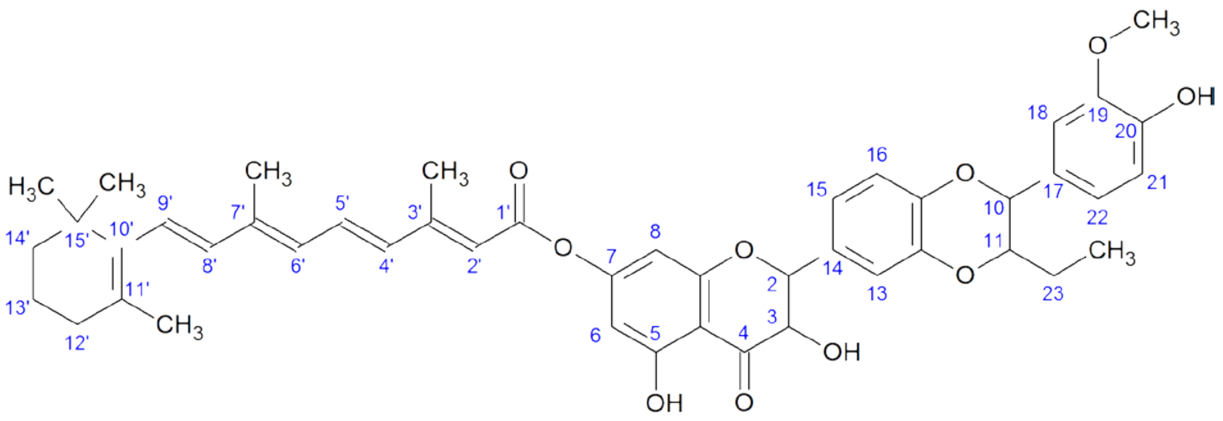

Atom #	δ_C	m	δ_H	n_H	m	$J_{H\text{-}H}$ [Hz]
**2**	82.60	d	5.254	1	d	11.5
**3**	71.67	d	4.775	1	dd	11.5, 6.3
**4**	199.34	s	-	-	-	-
**4a**	104.56	s	-	-	-	-
**5**	161.85	s	-	-	-	-
**6**	102.86	d	6.393	1	d	2.0
**7**	158.06	s	-	-	-	-
**8**	101.63	d	6.362	1	d	2.0
**8a**	161.68	s	-	-	-	-
**10**	75.74	d	4.928	1	d	7.9
**11**	77.92	d	4.172	1	ddd	7.9, 4.7, 2.7
**12a**	142.88	s	-	-	-	-
**13**	116.45	d	7.133	1	d	2.0
**14**	129.82	s	-	-	-	-
**15**	120.91	d	7.011	1	dd	8.3, 2.0
**16**	116.38	d	6.947	1	d	8.3
**16a**	143.92	s	-	-	-	-
**17**	127.38	s	-	-	-	-
**18**	111.66	d	7.007	1	d	1.9
**19**	147.51	s	-	-	-	-
**20**	146.92	s	-	-	-	-
**21**	115.24	d	6.805	1	d	8.1
**22**	120.36	d	6.859	1	dd	8.1, 1.9
**23**	60.09	t	3.547	1	ddd	12.3, 4.9,2.7
			3.350	1	ddd	12.3,5.9, 4.7
**3-OH**	-	-	5.944	1	d	6.3
**5-OH**	-	-	11.691	1	s	-
**19-MeO**	55.61	q	3.778	3	s	-

**20-OH**	-	-	9.108	1	s	-
**23-OH**	-	-	4.922	1	dd	5.9, 4.9
**1'**	163.44	s	-	-	-	-
**2'**	116.03	d	6.057	1	s	-
**3'**	156.46	s	-	-	-	-
**4'**	134.45	d	6.519	1	d	15.1
**5'**	132.91	d	7.195	1	dd	15.1, 11.7
**6'**	129.63	d	6.283	1	d	11.7
**7'**	140.50	s	-	-	-	-
**8'**	136.71	d	6.206	1	d	16.1
**9'**	128.55	d	6.326	1	d	16.1
**10'**	137.14	s	-	-	-	-
**11'**	129.74	s	-	-	-	-
**12'**	32.55	t	2.01 ^{H}	2	m	-
**13'**	18.61	t	1.578	2	m	-
**14'**	39.10	t	1.444	2	m	-
**15'**	33.77	s	-	-	-	-
**3'-Me**	13.85	q	2.349	3	d	0.8
**7'-Me**	12.61	q	2.018	3	s	-
**11'-Me**	21.42	q	1.697	3	s	-
**15'-Me**	28.71	q	1.022	3	s	-

^{H} - HSQC readout
